# Farewell to Bernard Witholt, A pioneering microbial biotechnologist

**DOI:** 10.1111/1751-7915.12293_4

**Published:** 2015-06-15

**Authors:** Kenneth N Timmis

**Affiliations:** Institute of Microbiology, Technical University BraunschweigSpielmannstr. 7, D-38106, Braunschweig, Germany

Bernard Witholt was one of the most foresighted, gifted and personable scientists I have ever known in a long academic career blessed by association with many exceptional scholars.

Despite coming from different branches of microbiology – Bernard was a biochemist, I a geneticist – we followed to some extent parallel research paths, working on microbial pathogenesis (e.g. enteric toxins), environmental biotechnology (e.g. degradation of organic pollutants), biocatalysis (e.g. hydrocarbon oxidations), bioplastics (PHA) and development of enabling experimental systems (e.g. *Pseudomonas* as a cell factory). We also shared young scholars – Marcel Wubbolts, Menno Kok, Auxi Prieto, Birgit Kessler and Sven Panke – to our great mutual benefit and pleasure. However, it was the creation of the European Environmental Research Organisation – the EERO – that really intensified our contacts and cemented our long-term association. Although this organization, created to promote top environmental research in Europe and conceived to be analogous to the European Molecular Biology Organisation, ultimately failed to acquire sustained funding from European governments, it succeeded initially in obtaining significant funds, and in promoting environmental research by providing a number of talented young scientists with long-term postdoctoral fellowships. Bernard, along with Sascha Zehnder, Willy Verstraete, Ivano Bertini, Ralf Hütter, Bernard Dixon and I formed the first EERO Council responsible for developing the basic concept and strategy, and for obtaining funding. While most of us were discussing about the best way to finance the EERO, Bernard and Sascha immediately cut to the chase and posed the rhetorical question: when you need money, where do you go? So they went to see a Dutch banker (and a few other key figures), returning with a commitment of about 5m guilders to serve as matching funds for funds raised in other countries! I am simplifying somewhat (sorry other Council Members!), but illustrating signature qualities of Bernard, namely his can-do attitude, infectious enthusiasm for projects he was convinced about and incredible powers of persuasion. Working with Bernard was exciting and supremely pleasurable: a true privilege.

As a scholar, Bernard was supremely a pioneer, combining great foresight with a rigorous biochemical approach in grasping new opportunities: he pioneered alkane degradation/oxidation and PHA production (petrochemical biorefineries, biofeedstocks, and bioplastics) years before they became hot topics. He made significant fundamental discoveries and developed proof-of-principle processes for industrial applications. And, as documented in the first tribute, he went the extra mile towards translation, by developing novel two-phase fermentation systems for reactions involving hydrophobic substrates or products, and by optimizing biocatalysis and bioproduction regimes and defining production-relevant parameters, so that he could convince potential industrial partners of the feasibility of the biotechnological processes he developed. He was also incredibly fertile in creating start-ups based on processes and ideas he developed. He was always original and creative, always at the front, and, in those rare cases where competition actually did exist, was generally well ahead. 

As a person, he was cosmopolitan, having roots in the Netherlands, Brazil (https://www.amherst.edu/alumni/classpages/1964/spk64/node/539153), the USA and finally Switzerland, but also a patriot with an abiding love of Holland, Dutch friends and the Dutch culture. He was a true gentleman: eloquent, cultivated, elegant, a bon viveur and gourmet. He was convivial and extremely entertaining, with a highly animated manner of articulating, a spontaneous humour and an infectious grin; it was pure pleasure to be in his company. He was a dedicated and successful rower and with Renske lived life to the full. 

He treated his illness as a biological activity worthy of study, was frankly open about it to friends and colleagues, and analysed his deteriorating biological parameters with academic detachment. He planned to have lunch with me in Bern only days before his death. His courage was impressive and exemplary.

It is impossible to list and illustrate all the manifold scholarly and personal qualities of Bernard in a short space: suffice it to say that they have enormously enriched academic and applied research, and inspired and moulded generations of young scholars who in the meantime have become successful inspiring leaders in their own right – the Witholt School. Bernard will not have realized it at the time, but his early work, with that of just a handful of other heavyweights, essentially jump-started the emerging field of microbial biotechnology and set high and durable benchmarks of originality, significance and rigour for the field. His group was a beacon. Bernard will be sorely missed by many. But his legacy, long established in his publications and in the Witholt school of scholars, will last as long as microbial biotechnology.

